# Relationship among Connectivity of the Frontal Aslant Tract, Executive Functions, and Speech and Language Impairment in Children with Childhood Apraxia of Speech

**DOI:** 10.3390/brainsci13010078

**Published:** 2022-12-31

**Authors:** Clara Bombonato, Emilio Cipriano, Chiara Pecini, Claudia Casalini, Paolo Bosco, Irina Podda, Michela Tosetti, Laura Biagi, Anna Maria Chilosi

**Affiliations:** 1 Department of Developmental Neuroscience, IRCCS Stella Maris Foundation, 56128 Pisa, Italy; 2 Department of Neuroscience, Psychology, Drug Research and Child Health (NEUROFARBA), 50139 Florence, Italy; 3 Laboratory of Medical Physics and Magnetic Resonance, IRCCS Stella Maris Foundation, 56128 Pisa, Italy; 4 Department of Physics, University of Pisa, 56127 Pisa, Italy; 5 Department of Education, Languages, Intercultures, Literatures and Psychology (FORLIPSI), University of Florence, 50121 Florence, Italy; 6 Parole al Centro Studio di Logopedia e Neuropsicomotricità, 16129 Genova, Italy

**Keywords:** childhood apraxia of speech, executive functions, frontal aslant tract

## Abstract

Childhood apraxia of speech (CAS) is a subtype of motor speech disorder usually co-occurring with language impairment. A supramodal processing difficulty, involving executive functions (EFs), might contribute to the cognitive endophenotypes and behavioral manifestations. The present study aimed to profile the EFs in CAS, investigating the relationship between EFs, speech and language severity, and the connectivity of the frontal aslant tract (FAT), a white matter tract involved in both speech and EFs. A total of 30 preschool children with CAS underwent speech, language, and EF assessments and brain MRIs. Their FAT connectivity metrics were compared to those of 30 children without other neurodevelopmental disorders (NoNDs), who also underwent brain MRIs. Alterations in some basic EF components were found. Inhibition and working memory correlated with speech and language severity. Compared to NoND children, a weak, significant reduction in fractional anisotropy (FA) in the left presupplementary motor area (preSMA) FAT component was found. Only speech severity correlated and predicted FA values along with the FAT in both of its components, and visual-spatial working memory moderated the relationship between speech severity and FA in the left SMA. Our study supports the conceptualization of a composite and complex picture of CAS, not limited to the speech core deficit, but also involving high-order cognitive skills.

## 1. Introduction

Childhood apraxia of speech (CAS) is a subtype of developmental motor speech disorder in which the precision and consistency of movements underlying speech are impaired in the absence of neuromuscular deficits, as currently defined by the American Speech–Language–Hearing Association [1]. It is reported that 2.4% of children with speech sound disorders may be diagnosed with CAS, with a higher prevalence in males [1,2]. The CAS core deficit involves the planning and/or programming of the spatiotemporal parameters of movement sequences [1]. According to the ASHA consensus criteria, three features are characteristic of CAS: (a) inconsistent errors on consonants and vowels during repeated productions of syllables or words, (b) lengthened and disrupted co-articulatory transitions between sounds and syllables, and (c) inappropriate prosody, especially in the realization of lexical or phrasal stress. These symptoms, together with a reduced phonetic inventory, multiple speech sound errors, and disfluency result in an effortful, unintelligible speech that has a negative impact on the children’s social communication and peer interactions. Children with CAS display altered speech timing and sequencing skills and show particular difficulties in dynamic transitions between articulatory postures and in combining smaller units of movement into larger ones. Difficulties in early oromotor and phono-articulatory aspects of speech acquisition in CAS may stem from weaker systematic mappings between articulatory gestures and their auditory effects [3]. Along with its isolated presentation, CAS usually co-occurs with language impairment (LI) [4,5,6], particularly in the expressive domain (grammar and lexicon).

### 1.1. Executive Functions

In addition to the motor speech core deficit, some studies have shown the presence of other areas of cognitive difficulty in children with CAS. Shriberg and colleagues suggested that CAS is a multilevel disorder in which both planning/programming (transcoding) and auditory-perceptual (encoding) deficits are involved, together with memory processes [7]. Moreover, the difficulty in working memory has been described on several levels (auditory coding, maintenance, and transcoding) [7,8] and seems to persist into adulthood [9]. A constellation of other functional deficits (phonological awareness and rapid naming) seems to characterize these children, together with learning difficulties at school-age, especially if the disorder is persistent and associated with language impairment [4,8,10,11,12]. Moreover, difficulties in nonverbal sequential functioning have been described [13,14,15], highlighting the presence of cognitive endophenotypes that support a broader conceptualization of the disorder. 

Executive functions (EFs) have also been called into question in association with developmental language impairment [16,17,18,19,20]. Early acquisition of good EFs represents a protective factor for the development and adaptation of human beings [21], given that EFs often have a greater influence than IQ and socioeconomic status in predicting quality of life [22]. There is a debate about the nature of executive functions. Some cognitive models conceptualize EFs as a unitary construct [23,24], but the idea that EFs can be fractionated into different—although interrelated—functions is supported by most accepted developmental cognitive models [21,22,25,26], claiming the presence of three core components—inhibition, updating working memory, and cognitive flexibility—which share a common purpose: the recruitment of attention and control over behavior in order to meet an adaptive goal. “Inhibition” refers to the deliberate control of prepotent responses and allows one to both resist temptations and impulsive actions (response inhibition) and to maintain focused attention by suppressing nonrelevant information (interference control). A lot of cognitive, behavioral, and emotional processes, such as abstract reasoning and self-regulation in affective and emotional contexts, require inhibitory control, which allows for more appropriate behaviors oriented to internal or external goals [27,28]. “Updating working memory” refers to the ability to actively and dynamically code, maintain, monitor, update, and manipulate incoming verbal or visual-spatial information [29,30,31]. Cognitive flexibility allows one to shift between mental sets and involves the ability to engage and disengage from different tasks, rules, or mental contents. It supports creative thinking and the capacity to solve problems in different ways or to see things from different perspectives. EFs develop from preschool-age to childhood and into adulthood [32,33,34], following the maturation of prefrontal circuitries and their connections [35]. 

### 1.2. Neuroanatomical Correlates of CAS

The frontal aslant tract is a brain white matter tract connecting the superior frontal gyrus (SFG), specifically the presupplementary motor area (preSMA), the supplementary motor area (SMA), and the lateral SFG to the pars opercularis and pars triangularis of the inferior frontal gyrus (IFG) and the anterior insula [36]. Over the last few years, research on the functional role of the FAT on speech and language processes has gained attention due to its well-known connections with “Broca’s area” [37]; with the preSMA and SMA regions, which have been associated with aphasia of the SMA [38]; and with impaired speech production [39]. In vivo FAT reconstruction is possible thanks to diffusion weighting imaging (DWI). Recent research studies have investigated the functional role of the FAT, reporting an involvement of this tract’s fibers with speech and language function in the left hemisphere, and an involvement of the right FAT in support of EFs [40]. Neuroimaging studies have demonstrated that regions connected through the FAT play a key role in expressive language and motor speech. In fact, the left IFG has been associated with controlled lexical and phonological selection and retrieval [41,42], and a lesion in this area seems to produce nonfluent aphasia symptoms [43]. Studies on the mapping of the left IFG in healthy and in clinical adults demonstrated the role of this cortical area in language and motor speech processing and in phonatory control [44,45]. Regions of the SMA and preSMA have been associated with high-order selection and execution in both speech and nonspeech domains [46,47], and a lesion in these areas can lead to motor and speech deficits, especially for volitional movements and speech [48,49]. The FAT is also associated with verbal fluency in persistent developmental stuttering [50,51,52] and in typical development [53,54], supporting its function in establishing preferred responses in the language [40] as well as the speech domains [55]. In a case report using intraoperative electrical stimulation combined with diffusion tensor imaging fiber tracking, the stimulation of the FAT induced speech arrest, followed by its recovery when the stimulation was ended [56]. In a larger retrospective study, 17 adult patients who underwent awake craniotomy for left frontal lobe glioma showed a wide array of language and motor speech alterations, including speech arrest, stuttering, and vocalizations when the posterior part of the fronto-striatal and the FAT subsystem were stimulated [57].The right IFG has also been identified as a region activated in executive function behaviors, especially in inhibitory control [58], with an impairment of the same function associated with lesions in this area [59,60]. Motor stopping behaviors are sustained by a direct pathway from the right IFG and the subthalamic nucleus [61,62,63,64]. However, the right preSMA and SMA also seem to play a role in inhibitory control in a more extended network, particularly in suppressing behaviors that conflict with a goal [65,66]. The right SMA has also been proven to be implicated in working memory, particularly in the active mental manipulation of information [67], as demonstrated by working memory deficits in patients with SMA lesions when compared with healthy controls. 

Alterations in some areas belonging to the network connected by the FAT were found in CAS [68]. In particular, three intra- and interhemispheric subnetworks showed a reduction of fractional anisotropy (FA) in the CAS group, as compared to controls. Subnetwork 1 concerned the temporal regions of the left hemisphere, the role of which had already been hypothesized in CAS [69,70,71,72,73]. Subnetwork 2 included intra- and interhemispheric connections, involving the left precuneus, the right supplementary motor area, the left cuneus, and the right cerebellum. The results are in agreement with previous studies that hypothesized the role of these regions in conceptual planning during lexical search [74] and in high-level integrated functions [75]. Subnetwork 3 included intrahemispheric connections among the right angular gyrus, the superior temporal gyrus, and the inferior occipital gyrus, pointing to bilateral language involvement in CAS [76]. 

### 1.3. Aim of the Study

The present study aimed to investigate the EF profiles of a group of children with CAS with comorbid LI, hypothesizing that the presence of deficits may contribute to defining the cognitive endophenotype of this disorder. The study starts from the consideration of the involvement of the FAT as a key pathway for two important functional circuits: one related to motor speech control, and the other to executive functions. The two circuits are typically examined separately although motor speech and EFs possibly rely on overlapping mechanisms. 

Moreover, given the presence of alterations of areas belonging to this circuit in CAS, a second aim of this study was to relate motor speech and the EF profile with structural connectivity information using diffusion MRI. We hypothesized that children with CAS may show impaired connectivity of the FAT, with that being more relevant in those with an alteration in the EF components. 

## 2. Materials and Methods

### 2.1. Participants

A total of 30 children with CAS and co-occurrent LI, diagnosed at the Neurolinguistic and Neuropsychological Unit of IRCCS Stella Maris, were recruited. The group underwent a full speech, language, and EF assessment, as well as MRI examination. 

The group of children with CAS included 6 girls and 24 boys. Children with CAS were aged between 4.3 years and 6.11 years (mean age = 6.6 years; *SD* = 0.7 years. All children with CAS were right-hand dominant except one child. The identification of patients with CAS was based on a comprehensive clinical and instrumental assessment [13], which is the standard clinical protocol for the assessment of complex neuropsychological and neurodevelopmental disorders adopted at the facility in which the study was conducted. Eligibility criteria required Italian as the only, or primary, language spoken at home, age at clinical evaluation ≥4 years, and the ability to complete full neurological and speech and language assessments. Exclusion criteria were orofacial structural abnormalities, audiological deficits, epilepsy, known neurological and neurometabolic disorders, dysarthria, and comorbid attention deficit/hyperactivity disorder, autism spectrum disorder, and/or developmental coordination disorder.

The diagnosis of CAS was carried out by a multidisciplinary team in accordance with the three aforementioned ASHA criteria (2007) and with any combination of at least 5 of the 10 points on Strand’s checklist [77], detectable across at least three contexts that varied in difficulty. The identification of the diagnostic features was based on formal testing and on the perceptual analysis of videorecorded speech samples by two independent observers with expertise in developmental motor speech disorders. A group of 30 children with no speech and language concerns and no other neurodevelopmental disorders (NoND (mean age = 6.5 years; *SD* = 2.6 years), who had undergone a brain MRI for various reasons (including headache, seizures during fever, strabismus, cataract, paroxysmal vertigo, and diplopia) was also recruited in order to compare FAT connectivity measures between the two groups. The brain MRIs of the NoND group, as well as their neurological examination, were unremarkable.

Written parental informed consent and child assent for participation in the study and data publication were obtained in all cases. The study was approved by the Regional Pediatric Ethics Committee (CEP) 19-03-2018/RF2016-02361560.

### 2.2. Procedures and Measures 

#### 2.2.1. Clinical Assessment

To rule out the presence of co-occurring complex neurodevelopmental disorders, all cases underwent standard neurological and psychiatric examination by a specialized team. *DSM-5* clinical diagnostic criteria and specific assessment procedures were applied. 

The cognitive nonverbal abilities of participants with CAS (mean = 103.23; *SD* = 12.89) were assessed by using the Wechsler Preschool and Primary Scale of Intelligence, Third Edition (WPPSI-III [78]). 

#### 2.2.2. Speech and Language Assessment

Speech and language profiles were analyzed by two independent observers through formal testing and evaluation of spontaneous productions. The assessment protocol included: 

(a) Parental report on the child’s early vocal behavior, speech, language, and early motor developmental milestones, as well as familial antecedents for oral/written language disorders. Family history was considered significant if one or more members of the nuclear family had a history of any type of speech-language and/or learning disorders.

(b) Speech tasks: assessment of phonetic inventory, speech inaccuracy, inconsistency, syllable omissions, and diadochokinetic rate (DDK). Since there are no standard scores from norm-referenced tests for these measures, data from 40 TD Italian children with a mean age of 4.7 years (*SD* 0.47 years) were used as a reference. Speech intelligibility was assessed through the Intelligibility in Context Scale [79], Italian version). 

(c) Language assessment: standardized language tests for receptive and expressive vocabulary and grammar. 

Detailed descriptions of the speech and language assessments are reported in a recent work [13].

In order to estimate the overall level of speech and language proficiency, two composite severity scores were calculated based on five speech and four language measures, provided by a speech therapist. Given that, depending on the children’s ages and degrees of impairment, different standardized language tests were administered, to calculate the language composite severity score, we assigned for each measure: 0 when normal (>25th percentile or z-scores > −0.67), 0.5 when delayed (percentile scores between 6th and 25th or z-scores between −1.56 and −0.67), and 1 when deficient (≤5th percentile or z scores < −1.65). The maximum language composite severity score was 4, and 5 was the maximum speech severity score. On the basis of the speech and language severity scores, the sample was divided into two subgroups: 0–2.5: low language severity; 3–4: high language severity (20 children and 10 children, respectively); 0–3: low speech severity; 3.5–5: high speech severity (12 children and 18 children, respectively).

#### 2.2.3. Executive Function Assessment

In order to obtain an overall evaluation aimed at the definition of a specific EF profile in children with CAS, an ad hoc evaluation protocol to investigate the different EF components was created, selecting tasks from standardized batteries for the Italian population, and taking into account the age range of the sample. The protocol consisted of the following tasks:

- *Draw a Circle* (FE-PS 2–6; [80]). The child is asked to inhibit the continuous motor response: the task requires tracing a circle with a finger on a white sheet of paper, adapting the execution speed to the examiner’s request.
- *Day and Night Stroop* (FE-PS 2–6; [80]). The test involves the inhibition of the verbal response by suppressing a preponderant response prompted by a stimulus. The inhibition concerns the ability to block an automatic response and to manage the conflict between two response operations associated with the same stimulus. Both time and accuracy are measured.
- *Flanker Task* (FE-PS 2–6; [80]). The test assesses interference management: The child must indicate the direction of the central stimulus in the presence of interfering stimuli, which can be oriented either in the same direction as the target (congruence) or in the opposite direction (incongruence). Both time and accuracy are measured.
- *Dimensional Change Card Sort* (FE-PS 2–6; [80]). This test, which recalls the paradigm of the Dimensional Change Card Sort (DCCS), assesses the capacity for cognitive flexibility, inhibitory control, and working memory: The child must classify a series of cards, first by color, then by shape, and finally according to the color if the card has a black border and according to the shape if the card does not have a black border.
- *Keep Truck* (FE-PS 2–6; [80]) The test aims to evaluate the organization of information in working memory: The child is shown images belonging to five categories. Before the beginning of the test, the child is asked to pay attention to a particular category. A series of six images belonging to different categories are then shown, and the child must name them out loud. At the end of each series, the child is asked to remember the last image belonging to the designated category.
- *Spin the Pots* (BAFE, [81]. A visual research task that evaluates the visual-spatial working memory: The examiner places a red token under each of the eight pots arranged on a tray. The tray is then covered with a cloth and rotated. The child is asked to remove the cloth from the tray and find, one at a time, the tokens placed under each pot.

Scores obtained by the children with CAS were compared with standardized normative scores [80], considering as “deficits” those scores falling below the 5th percentile; as “immature” those scores below the 25th percentile; and as within the “normal” range those scores higher than the 25th percentile. In order to estimate the severity level of each EF component, each measure was assigned a score of 2 when normal (>25th percentile) and a score of 1 when deficient or delayed (<25th percentile). 

#### 2.2.4. Imaging Protocols

MRI data were acquired with a GE (General Electric Medical Systems, Chicago, IL, USA) HDxt 1.5T Signa MRI system at IRCCS Stella Maris Foundation. The protocol included: (1) a 3D T1-weighted structural sequence (3D BRAVO) with 1 mm isotropic resolution (time of repetition (TR)/time of echo (TE) = 12.37/5.18 ms; flip angle (fa) = 13°; field of view (FoV) = 256 mm × 256 mm; matrix = 256 × 256; slice thickness = 1 mm); (2) a diffusion weighted imaging acquisition (DWI), by using a 2D single-shot spin-echo EPI sequence with a 3 mm isotropic resolution (TR/TE = 13,000/115.8 ms; fa = 90°; FoV = 240 mm × 240 mm; matrix = 80 × 80; slice thickness = 3 mm), including 30 noncollinear encoding directions with a b-value of 1000 s/mm^2^, and one additional volume without diffusion gradients (b = 0 s/ mm^2^). 

#### 2.2.5. MRI Analysis and Postprocessing

A total of 2 out of the 30 children with CAS were excluded from the MRI analysis because of excessive motion. The 3D T1-weighted images were processed using FreeSurfer [82]. FreeSurfer was used for the preprocessing workflow for the structural MRI data to extract the white matter (WM), gray matter (GM), subcortical GM, and cerebrospinal fluid (CSF) structures [83]. 

The preprocessing of the DWI data was performed using FSL 6.0.4 [84] in particular for applying the corrections for head motion, induced eddy current, and EPI distortion. After preprocessing, the fractional anisotropy (FA) was extracted for each subject. FA is an invariant measure of the degree of diffusion anisotropy reflecting white matter integrity and varies between 0 and 1.

For the tracts of interest, their reconstructions were performed using the constrained spherical deconvolution (CSD) technique implemented in the MRtrix package [85]. The iFOD2 algorithm that facilitates more-accurate fiber reconstruction in heavily curved regions was used with a maximum selected number of streamlines of 10,000 [86]. In order to correct and increase the anatomical plausibility of the reconstructed fibers, the anatomically-constrained tractography (ACT) method was applied by using the 5-tissue-type (5TT) images obtained via FreeSurfer segmentation, removing streamlines that are anatomically unfeasible [87]. For each tract, according to the literature, we manually identified specific regions of interest (ROI), to be used as a seed, inclusion, or exclusion region in tractography reconstruction. 

The identified tract of interest was the frontal aslant tract (FAT), divided into supplementary motor area (SMA) and presupplementary motor area (preSMA) components. For both the SMA and preSMA components, a seed ROI was placed in the axial plane at the level of the inferior frontal gyrus pars opercularis (IFGop). The SMA-inclusion ROI was defined as rostral to the primary motor cortex and caudal to the vertical commissure anterior (VCA) line. The preSMA inclusion ROI was defined as rostral to the VCA line and caudal to the virtual line, passing through the genu of the corpus callosum [88]. 

Finally, the mean FA along each FAT component was calculated.

#### 2.2.6. Statistical analyses

In order to compare the mean FA values along the FAT between CAS and TD children, ANCOVA analyses were performed using age, sex, number of tracts, and the tract volume as covariates. Descriptive and inferential statistics were conducted using Statistical Package for Social Science 2022, version 28.0.1.0 (142) (SPSS, IBM Corporation, Armonk, NY, USA). First, data were analyzed to describe the distribution and the profile of the scores on the EF, speech, and language tasks. In order to identify the EF components most impaired in our sample, a nonparametric Friedman analysis was conducted. Point-biserial correlation analyses were used to investigate the relations between each EF task, speech and language severity, and FA values along the preSMA and SMA components of the FAT. On the basis of the correlation results, linear regression was used to determine the variance of FAT FA values explained by speech severity. Finally, to verify the interaction between speech severity and EF deficit on each task on the FA values of the FAT, a moderator model (Model 1; [89]) was run using PROCESS v 4.0 SPSS. Regression analyses based on 5000 bootstrap samples were used to estimate path coefficients and confidence intervals for the regression equations [89].

## 3. Results

### 3.1. Speech Profile

Compared to what is expected in the typical population of the same age, the phonetic inventories of children with CAS were markedly reduced, with a mean number of consonants of 12.7 (*SD* = 4.09), out of 21 consonantal sounds assessed. The mean percentage of inaccurate speech productions in a single-word naming task was 61%, with a rating of 24% of inconsistency errors in the same task. On the McLeod and colleagues intelligibility scale [79], modified for the Italian language, the average score was 2.32 (*SD* = 0.87), thus showing a severely altered level of intelligibility as perceived by the communication partners in spontaneous production contexts. Concerning the DDK rate, 24 children out of 30 were able to repeat the three-syllable nonword sequence/pataka/over 20 s. Their mean rate was significantly slower (number of repetitions: M = 15.13, *SD* = 4.01) compared to the reference data (number of repetitions: M = 25, *SD* = 4.7). The speech profile results are summarized in Table 1. 

Speech signs of CAS that were more frequently (80% or greater) detectable across the whole sample were: inconsistent productions, difficulties in transitioning from one speech movement to another, errors with vowels, reduced consonantal repertoire, atypical phonological processes, syllable omissions, increasing difficulties in longer units, dysprosody, and a slow and/or scanned speech rate.

### 3.2. Language Profile

Concerning receptive grammar, 80% of the children with CAS had normal (>25th percentile) or borderline (between 25th and 6th percentile) scores, whereas 20% of the children showed a deficit (scores <5th percentile). On expressive grammar evaluation, 87% of the children scored below the 5th percentile. The expressive lexicon was deficient in 17% of the children. The language profile results are summarized in Table 2.

**Table 2:** Language profile results of children with CAS. Assessment measures are reported.

Language Assessment Protocol of CAS Group	% <5° Percentile/ <−1.65 z Score	Assessment Measures
Expressive Grammar	87%	Grid for the Analysis of Spontaneous Speech (GASS [90])
Receptive Grammar	20%	TCGB, Test di Comprensione Grammaticale per Bambini (Grammar comprehension test for children) [91] TROG-2 Test for Reception of Grammar, Version 2. [92]
Receptive vocabulary	10%	Test Fonolessicale (TFL [93],) and/or Peabody Picture Vocabulary Test (PPVT-R [94]), depending on the child’s age and on the severity of the disorder
Expressive vocabulary	17%	Test Fonolessicale (TFL [93] and/or One-Word Picture Vocabulary Test [95] depending on the child’s age and on the severity of the disorder

### 3.3. EF Profile

**Figure 1:**
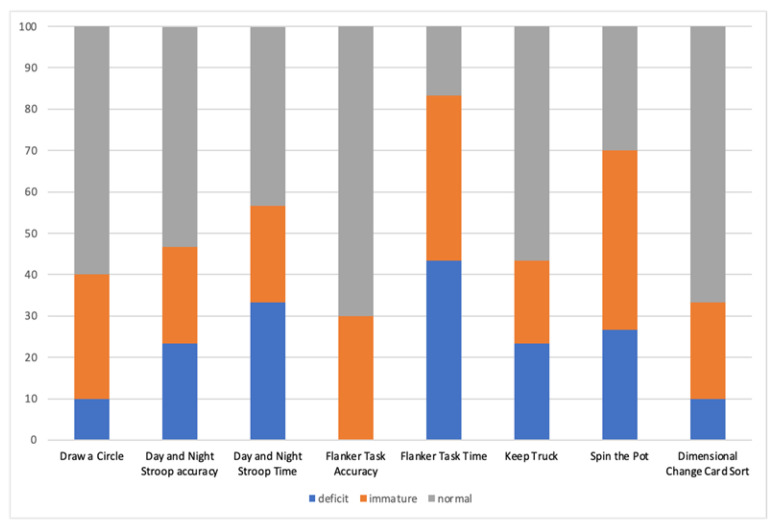
Frequency distribution of performance in each EF task in children with CAS.

The Friedman test showed that the distribution of normal vs. impaired scores (immature + deficit) significantly differed among tasks (chi-squared (30) = 29.421; *p* < 0.001). As described below (Figure 1), the highest percentage of impaired scores was found in the Flanker Task and Spin the Pot task.

In regard to response inhibition, in a task in which a motor response is required (Draw a Circle), 10% of the children showed a deficit and 30% had an immature performance, while 60% of the sample scored within the normal range. In a visual-verbal task (Day and Night Stroop), in the accuracy parameter, 23.4% of the sample obtained deficient scores, 23.3% demonstrated an immature performance, and 53.3% demonstrated a normal performance for their ages. Regarding the time parameter of the same task, 33.4% had deficient scores, 23.3% immature scores, and 43.3% normal scores. With regard to the ability to control interference, as assessed with a visual-spatial task (the Flanker Task), in the accuracy parameter, no child had a deficient score, 30% had immature scores, and 70% showed normal scores. Conversely, 43.3% had deficient scores for the time parameter, 40% showed an immature performance, and only 16.7% had a performance within the normal range. Concerning updating in working memory, in a visual-verbal task (Keep Truck), 23.3% of the sample obtained deficient scores, 20% showed an immature performance, and 56.7% scored within the norm for their ages. In a visual-spatial task (Spin the Pot), 26.7% scored in the deficit area, 43.3% obtained scores within the immaturity range, and 30% scored in the normal range. With regard to cognitive flexibility, in a visual-conceptual task (Dimensional Change Card Sort), 10% of the children with CAS obtained deficient scores, 23.3% showed an immature performance, and 66.7% scored within the norm for their ages. 

The EF profile results are summarized in Table 3. 

Covarying for age, gender, and handedness, language severity significantly correlates with accuracy on the Flanker Task (r = 0.613; *p* < 0.005) and Keep Truck task (r = 0.627; *p* < 0.005), while no significant correlation emerged between speech severity and EF measures.

### 3.4. FAT Reconstruction, Analysis, and Relations with Speech and Language Profile 

**Figure 2:**
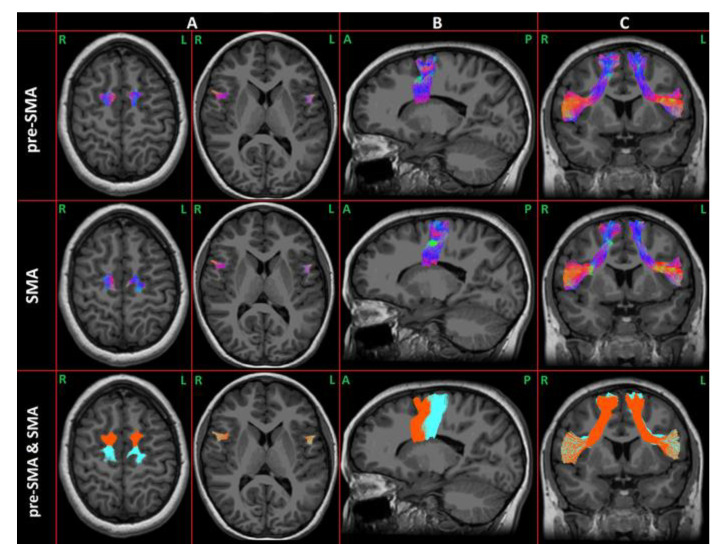
Example of the FAT: The first two rows represent the preSMA and SMA components. The direction of the tract is coded using RGB for the XYZ direction: red indicates the left–right direction, green the anterior–posterior direction, and blue the superior–inferior one. In the last row are overlapped both the FAT components (orange for preSMA and light-blue for SMA). Panel (**A**) shows the top and the bottom of the tracts in the axial plane. In panels (**B**,**C**), the projections of the FAT in the sagittal and coronal planes are respectively represented.

Each component of the FAT was extracted from each hemisphere of each CAS and TD subject. An example case is shown in Figure 2.

The mean FA values along the FAT were compared between the CAS and TD children (see Table 4). A significant difference was found for the left component of the FAT preSMA (*p* < 0.05). Furthermore, this difference did not survive multiple comparison correction.

**Figure 3:**
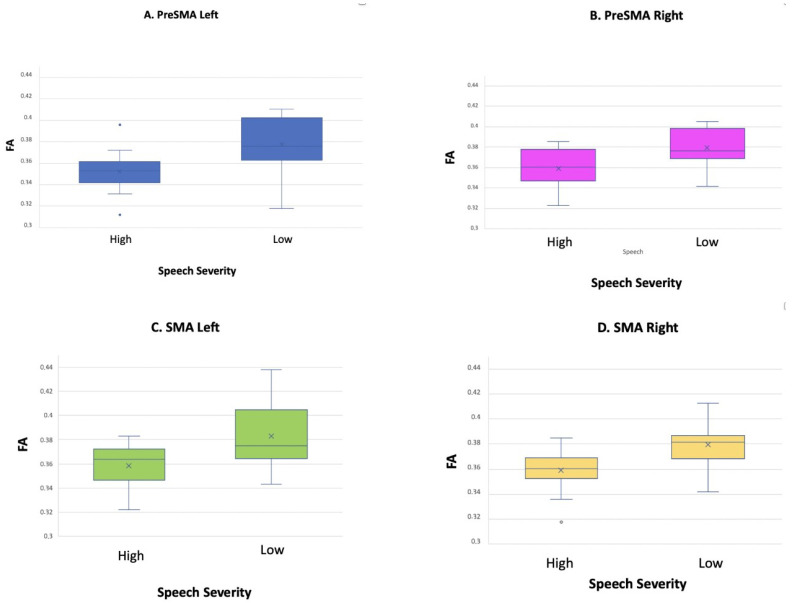
Significant point-biserial correlation between Speech Severity and FA along the FAT in the preSMA component in left hemisphere (**A**), FA along the FAT in the preSMA component in the right hemisphere (**B**), FA along the FAT in the SMA component in the left hemisphere (**C**)**,** and FA along the FAT in the SMA component in the right hemisphere (**D**).

Significant correlations emerged between speech severity and FA values along the preSMA component in the left hemisphere (r = 0.470; *p* < 0.05) (Figure 3A), in the right hemisphere (r = 0.519; *p* < 0.01) (Figure 3B), between speech severity and FA values along the SMA component in the left hemisphere (r = 0.481; *p* < 0.05) (Figure 3C), and in the right hemisphere (r = 0.557; *p* < 0.01) (Figure 3D), thus indicating a reduced white matter integrity in each FAT component in correspondence with greater speech severity. In order to confirm the expected changes on FA values along the FAT based on the severity of the disorder, linear regression analysis of speech severity on the FAT FA was conducted. Speech severity significantly predicted FA variance for each FAT component, with a percentage of explained variance ranging from 21% to 27% (preSMA left: R^2^ = 0.24; β = 0.49; *p* < 0.01; preSMA right: R^2^ = 0.21; β = 0.46; *p* < 0.05; SMA component: SMA left R^2^ = 0.22; β = 0.48; *p* < 0.05; SMA right: R^2^ = 0.27; β = 0.52; *p* < 0.01). 

No significant correlations emerged between the FA value of the FAT in either the SMA or the preSMA components, nor in the language severity score, nor in the EF tasks.

### 3.5. Moderation Analysis

A moderation test describes how the interaction between two different variables can influence the occurrence of an effect. In order to investigate whether EFs moderate the predictive role of speech severity on the FAT FA, a moderator analysis (Model 1, [89]) was conducted for each EF and FAT component. The moderation analysis aimed to test the hypothesis that working memory, one of the most impaired EF components in our sample, and reported in the literature to be deficient in CAS [7,8,9], may moderate the relationship between speech severity and FA value of the FAT SMA component, which underlies several higher-order control functions during speech production. Its role is particularly relevant in complex speech activities [96], and its efficiency might be affected by the severity of the speech disorder, as well as by domain-general control difficulties in the continuous updating of motor plans.

The moderation analyses showed that visual-spatial working memory significantly moderated the predictive role of speech severity on the FA value of the left FAT SMA component (B *≠* 0, SS × VS-WM *p* < 0.05; see Table 5); the R-squared increase due to the interaction was also significant (R^2^ = 0.10, F (1, 24) = 4.45, *p* < 0.05).

**Figure 4:**
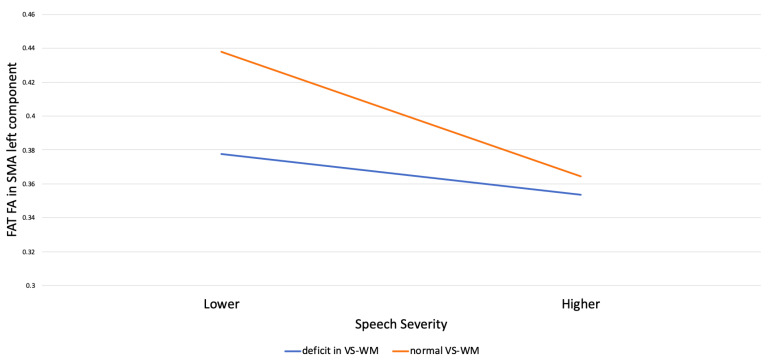
Interaction between severity of speech and Visual-Spatial Working Memory (VS-WM) on the FA value of the FAT in the left SMA component.

The estimation of conditional effect of speech severity on the FA value along the FAT in the SMA left component at two levels (deficit/normal) of visual-spatial working memory showed that speech severity significantly affected the FA value along the FAT in the left SMA component at both visual-spatial working memory levels, although with a larger effect size for normal (*β* = −0.844) compared to deficient (*β* = −0.507) working memory ability, (Figure 4).

## 4. Discussion

While a relatively large body of literature is dedicated to the study of the speech characteristics of children with CAS, only recently has the neuropsychological profile started to gain attention from researchers. The existing literature on CAS reports a high rate of co-occurring cognitive–linguistic weaknesses in this population [4,13,14,97], and in our sample, children also showed co-occurring language impairments, mainly involving expressive grammar. However, less is known about the relationship of co-occurring deficits with the speech features that are central to a CAS diagnosis. The current study examined both the direct effect and the interaction of the core deficit of the disorder (the severity of speech) with higher-order cognitive processes (the executive functions) on brain connectivity in a fiber tract functionally relevant to the disorder, the frontal aslant tract (FAT). The main reason to study EFs in CAS was based on the need to investigate specific neuropsychological processes potentially related to the clinical manifestations. 

The results of the present study showed the presence of a complex functional profile, characterized by difficulties not only in the speech domain, but also in specific EF components. Visual-spatial working memory, in particular, appears to be the most frequently compromised component in terms of accuracy. This result extends what was found by previous studies on phonological working memory [7,8] and also to the visual-spatial processing mode, configuring the working memory impairment as a general domain deficit independent of the information processing mode (verbal vs. visual). The alteration found in a working memory task, in which information updating is highly required, demonstrates that, in these children, not only the articulatory repetition mechanism is compromised [7], but also the updating and the active manipulation of information are altered. The former contributes to the ability to retain information in the phonological circuit by keeping it “active” [98], while updating allows the modification of the contents of the memory to accommodate new input [99].

Moreover, response inhibition and interference control were found to be frequently compromised in the speed parameter, the latter being deficient or immature in 83.3% of the sample, despite not being frequently compromised in the accuracy parameter, in which only 30% of the sample had a poor or immature performance. This adds to the literature that shows a general slowdown and slower performances in simple reaction time tasks in children with CAS, as compared to their peers with typical development or with other speech–sound disorders [14,100]. Our evidence suggests that, to achieve a satisfactory level of accuracy in suppressing interfering stimuli, children with CAS require a longer processing time. This could suggest and help to explain why these children require intense practice with numerous repetitions to achieve their treatment objectives [101,102,103,104] although it is not known how much practice they would require to reach the same processing times as their peers. Furthermore, the severity of the co-occurring language disorder was found to correlate with the accuracy of interference control, as also shown in children with isolated language disorder [105]. Moreover, language severity scores correlated with verbal working memory update scores, pointing to the influence of linguistic difficulties, not only with short-term repetition or processing in phonological working memory [16,17,106,107,108], but also with the semantic-lexical updating of information.

Therefore, with regard to the EF profile, multiple alterations are confirmed mainly in the basic components of EFs, such as inhibition and working memory, rather than in more complex components related to categorical abstraction and cognitive flexibility. Since the sample for our study was of preschool age, it is possible that the assessment tools were more sensitive to grasping difficulties in the components of EFs that develop early, rather than in those that tend to emerge later and require a wider developmental period [21,109].

These results are clinically relevant as it is not commonplace to evaluate the profile of executive functions in children with CAS, while the investigation of these processes could provide relevant information for the definition of the functional profile.

Furthermore, EF impairment showed a complex pattern of relation with speech severity and neurofunctional findings. Through a diffusion MRI specific protocol, each component of the FAT was extracted. White matter integrity was compared between CAS and TD children, revealing a significant reduction in FA values in the left component of the FAT preSMA. Speech severity, but not language severity, correlated and predicted FA values along the FAT in both of its investigated components (the SMA and preSMA), and EF impairment moderated this relation. In particular, a significant role for visual-spatial working memory in moderating the relationship between speech severity and FA value along the FAT in the left SMA component was found. The relationship is significant for both deficient and normal levels of visual-spatial working memory, the latter with a larger effect size. Therefore, especially in conditions of lower speech severity, good visual-spatial working memory skills are associated with a greater integrity of the white matter in this area.

This result is of particular importance, as it underlines, on the one hand, the importance of the evaluation of executive functions in defining the functional profile of CAS and, on the other, it stimulates reflection on rehabilitation. Fractional anisotropy increase has been associated with neuroplastic effects induced by processes connected with learning [110,111,112], and in particular, in CAS, an improvement in speech has been demonstrated parallel to the increase in FA in the left ventral and right dorsal corticobulbar tracts following treatment focused on speech motor control, thus supporting the treatment-induced neuroplastic effect [113]. Following Fiori and colleagues’ suggestion [113] and, given that the role of the FAT has never been investigated in CAS but has been studied as a functionally relevant fiber pathway both in speech [114] and in executive functions [40], we decided to examine the role of these two processes and their functional relationship with the FAT in CAS. Our results support the presence of a direct role of speech severity on white matter integrity in the SMA component of the FAT, an area associated with selection and execution in the production of words and of oral motor gestures [46,47]. However, new light has been shed on the role of higher-order skills, such as executive functions and, especially, on working memory and inhibition. A deficit in inhibiting the previous motor plans and updating new sequences [115], in fact, could affect the ability to program and plan the space-time parameters of movement sequences. Although further studies are necessary to confirm this assumption, we could argue that the enhancement of these abilities within a specific treatment could lead to a “far transfer effect” on the mitigation of the clinical manifestations of the disorder and on the generalization of learning, also verified at the neurofunctional level.

A limitation of the present study is the small sample size, mainly due to the low incidence of idiopathic CAS [116]. Additional research with a larger sample is required to substantiate our results. A further limitation is the absence of a control group undergoing both the EF and speech and language evaluation protocol and the MRI acquisition, as the CAS children did. Another limitation was the relative homogeneity of our participants with CAS, who were characterized by a high rate of co-occurring language impairments. In order to verify the generalizability of our findings, a group with higher variability across their language skills should be included in further studies. Finally, longitudinal and pre/posttreatment studies could allow us to better understand the long-term consequences of the relationship between the core deficit of speech and executive functions in CAS.

## 5. Conclusions

In conclusion, our study extends the understanding of CAS, a persistent and severe developmental motor speech disorder, as a composite and complex condition, frequently involving higher order cognitive skills, such as EFs. In particular, the alterations in control inhibition and updating in working memory may play a critical role in maintaining the severity and persistence of the disorder over time. The results obtained underline the importance of a comprehensive multidisciplinary assessment, which becomes mandatory in order to provide a more in-depth characterization of the disorder and define the most appropriate therapy interventions. We believe that the present findings pave the way to future studies which consider the effect of higher-order skills empowerment in specific disorders in order to identify, on the one hand, the preferential treatment for each specific condition, and, on the other, which are the specific characteristics of the different treatments that allow an effective improvement of clinical symptoms.

## Figures and Tables

**Table 1:** Speech profile results of children with CAS. Reference data are reported.

Speech Assessment Protocol	CAS Group	Reference Data
Phonetic inventory	*M* = 12.7 (*SD* = 4.09)	40 TD children, mean age = 4.7 years (*SD* = 0.47 years) Mean number of phonemes: 19.2 (*SD* = 0.9)
Word Inaccuracy	61%	40 TD children, mean age = 4.7 years (*SD* = 0.47 years) Mean percentage of inaccurate productions: 8.8% (*SD* = 10.7)
Inconsistent errors on consonants and vowels	24%	40 TD children mean age = 4.7 (*SD* = 0.47 years) Mean percentage of inconsistent errors: 0.4% (*SD* = 1.3)
DDK rate (maximum performance task)	*M* = 15.13 (*SD* = 4.01)	40 TD children (mean age = 4.7 years (*SD* = 0.9 years) Mean number of repetitions: 23.18 (*SD* = 4.5)
Intelligibility	*M* = 2.32 (*SD* = 0.87).	Qualitative rating scale ranging from 5 to 1 (5 = always, 4 = usually, 3 = sometimes, 2 = rarely, 1 = never intelligible)

**Table 3:** EF profile results of children with CAS.

EF Assessment Protocol of CAS Group	% <5° Percentile	% <10° Percentile	% >25° Percentile
Motor Response Inhibition (Draw a Circle)	10%	30%	60%
Visual-Verbal Response Inhibition (Day and Night Stroop, accuracy)	23.4%	23.3%	53.3%
Visual-Verbal Response Inhibition (Day and Night Stroop, time)	33.4%	23.3%	43.3%
Visual-Spatial Control Interference (Flanker Task, accuracy)	0%	30%	70%
Visual-Spatial Control Interference (Flanker Task, time)	43.3%	40%	16.7%
Visual-Verbal Updating (Keep Truck)	23.3%	20%	56.7%
Visual-Spatial Updating (Spin the Pot)	26.7%	43.3%	30%
Visual Cognitive Flexibility (Dimensional Change Card Sort)	10%	23.3%	66.7%

**Table 4:** Mean, Standard Deviation (SD), F, and *p*-value of the ANCOVA test for FA values of FAT components in CAS and TD children. * = statistically significant difference between groups (*p* < 0.05).

	CAS Mean (*SD*)	TD Mean (*SD*)	*F*	*p*
FA-left preSMA FAT	0.36 (0.02)	0.39 (0.02)	5.93	0.02 *
FA-right preSMA FAT	0.37 (0.02)	0.37 (0.03)	0.01	0.90
FA-left SMA FAT	0.37 (0.02)	0.39 (0.02)	2.79	0.10
FA-right SMA FAT	0.37 (0.02)	0.37 (0.03)	1.71	0.19

**Table 5:** Regression coefficients for moderation analysis on the FA value of the FAT in the left SMA component, including severity of speech as a predictive variable and visual-spatial working memory as a moderating variable.

	*B*	*S.E.*	*t*	*p*	*95% CI*
Speech severity (SS)	−0.025	−0.03	−0.89	0.38	−0.015–0.064
Visual-spatial working memory (VS-WM)	−0.039	0.03	−1.33	0.02	0.076–0.142
SS × VS-WM	0.05	0.02	2.11	<0.05	−0.072–−0.029

## Data Availability

The data presented in this study are available on request from the corresponding author.

## References

[1] ASHA—American Speech-Language-Hearing Association (2007). Technical Report on Childhood Apraxia of Speech.

[2] Shriberg L.D., Kwiatkowski J., Mabie H.L. (2019). Estimates of the Prevalence of Motor Speech Disorders in Children with Idiopathic Speech Delay. Clin. Linguist Phon..

[3] Maassen B., Nijland L., Terband H., Maassen B., van Lieshout P. (2010). Developmental Models of Childhood Apraxia of Speech. Speech Motor Control. New Developments in Basic and Applied Research.

[4] Lewis B.A., Freebairn L.A., Hansen A.J., Iyengar S.K., Taylor H.G. (2004). School-Age Follow-up of Children with Childhood Apraxia of Speech. Lang. Speech Hear. Serv. Sch..

[5] Chilosi A.M., Lorenzini I., Fiori S., Graziosi V., Rossi G., Pasquariello R., Cipriani P., Cioni G. (2015). Behavioral and Neurobiological Correlates of Childhood Apraxia of Speech in Italian Children. Brain Lang..

[6] Chilosi A.M., Podda I., Ricca I., Comparini A., Franchi B., Fiori S., Pasquariello R., Casalini C., Cipriani P., Santorelli F.M. (2022). Differences and Commonalities in Children with Childhood Apraxia of Speech and Comorbid Neurodevelopmental Disorders: A Multidimensional Perspective. J. Pers. Med..

[7] Shriberg L.D., Lohmeier H.L., Strand E.A., Jakielski K.J. (2012). Encoding, Memory, and Transcoding Deficits in Childhood Apraxia of Speech. Clin. Linguist. Phon..

[8] Lewis B.A., Avrich A.A., Freebairn L.A., Taylor H.G., Iyengar S.K., Stein C.M. (2011). Subtyping Children With Speech Sound Disorders by Endophenotypes. Top. Lang. Disord..

[9] Kenney M.K., Barac-Cikoja D., Finnegan K., Jeffries N., Ludlow C.L. (2006). Speech Perception and Short Term Memory Deficits in Persistent Developmental Speech Disorder. Brain Lang..

[10] Lewis B.A., Freebairn L.A., Taylor H.G. (2000). Academic Outcomes in Children with Histories of Speech Sound Disorders. J. Commun. Disord..

[11] Lewis B.A., Shriberg L.D., Freebairn L.A., Hansen A.J., Stein C.M., Taylor H.G., Iyengar S.K. (2006). The Genetic Bases of Speech Sound Disorders: Evidence from Spoken and Written Language. J. Speech Lang. Hear. Res..

[12] Smith S.D., Pennington B.F., Boada R., Shriberg L.D. (2005). Linkage of Speech Sound Disorder to Reading Disability Loci. J. Child. Psychol. Psychiatry.

[13] Bombonato C., Casalini C., Pecini C., Angelucci G., Vicari S., Podda I., Cipriani P., Chilosi A.M., Menghini D. (2022). Implicit Learning in Children with Childhood Apraxia of Speech. Res. Dev. Disabil..

[14] Iuzzini-Seigel J. (2021). Procedural Learning, Grammar, and Motor Skills in Children With Childhood Apraxia of Speech, Speech Sound Disorder, and Typically Developing Speech. J. Speech Lang. Hear. Res..

[15] Nijland L., Terband H., Maassen B. (2015). Cognitive Functions in Childhood Apraxia of Speech. J. Speech Lang. Hear. Res..

[16] Montgomery J., Magimairaj B., Finney M. (2009). Working Memory and Specific Language Impairment: An Update on the Relation and Perspectives on Assessment and Treatment. Am. J. Speech-Lang. Pathol./Am. Speech-Lang.-Hear. Assoc..

[17] Duinmeijer I., de Jong J., Scheper A. (2012). Narrative Abilities, Memory and Attention in Children with a Specific Language Impairment. Int. J. Lang. Commun. Disord..

[18] Henry L.A., Messer D.J., Nash G. (2012). Executive Functioning in Children with Specific Language Impairment: Executive Functioning and SLI. J. Child Psychol. Psychiatry.

[19] Bishop D.V.M., Norbury C.F. (2005). Executive Functions in Children with Communication Impairments, in Relation to Autistic Symptomatology. 2: Response Inhibition. Autism.

[20] Marton K. (2008). Visuo-Spatial Processing and Executive Functions in Children with Specific Language Impairment. Int. J. Lang. Commun. Disord..

[21] Diamond A. (2013). Executive Functions. Annu. Rev. Psychol..

[22] Diamond A., Ling D.S. (2016). Conclusions about Interventions, Programs, and Approaches for Improving Executive Functions That Appear Justified and Those That, despite Much Hype, Do Not. Dev. Cogn. Neurosci..

[23] Baddeley A. (2012). Working Memory: Theories, Models, and Controversies. Annu. Rev. Psychol..

[24] Kane M.J., Engle R.W. (2002). The Role of Prefrontal Cortex in Working-Memory Capacity, Executive Attention, and General Fluid Intelligence: An Individual-Differences Perspective. Psychon. Bull. Rev..

[25] Friedman N.P., Miyake A. (2017). Unity and Diversity of Executive Functions: Individual Differences as a Window on Cognitive Structure. Cortex.

[26] Miyake A., Friedman N.P., Emerson M.J., Witzki A.H., Howerter A., Wager T.D. (2000). The Unity and Diversity of Executive Functions and Their Contributions to Complex “Frontal Lobe” Tasks: A Latent Variable Analysis. Cogn. Psychol..

[27] Zelazo P.D., Qu L., Müller U. (2005). Hot and Cool Aspects of Executive Function: Relations in Early Development. Young Children’s Cognitive Development: Interrelationships among Executive Functioning, Working Memory, Verbal Ability, and Theory of Mind.

[28] Zelazo P.D., Müller U., Goswami U. (2002). Executive Function in Typical and Atypical Development. Handbook of Childhood Cognitive Development.

[29] Baddeley A. (2003). Working Memory: Looking Back and Looking Forward. Nat. Rev. Neurosci..

[30] Baddeley A.D., Hitch G.J. (1994). Developments in the Concept of Working Memory. Neuropsychology.

[31] Smith E.E., Jonides J. (1999). Storage and Executive Processes in the Frontal Lobes. Science.

[32] Hughes C., Ensor R., Wilson A., Graham A. (2009). Tracking Executive Function Across the Transition to School: A Latent Variable Approach. Dev. Neuropsychol..

[33] Lehto J.E., Juujärvi P., Kooistra L., Pulkkinen L. (2003). Dimensions of Executive Functioning: Evidence from Children. Br. J. Dev. Psychol..

[34] Somerville L.H., Casey B.J. (2010). Developmental Neurobiology of Cognitive Control and Motivational Systems. Curr. Opin. Neurobiol..

[35] Gilbert S.J., Burgess P.W. (2008). Executive Function. Curr. Biol..

[36] La Corte E., Eldahaby D., Greco E., Aquino D., Bertolini G., Levi V., Ottenhausen M., Demichelis G., Romito L.M., Acerbi F. (2021). The Frontal Aslant Tract: A Systematic Review for Neurosurgical Applications. Front. Neurol..

[37] Tremblay P., Dick A.S. (2016). Broca and Wernicke Are Dead, or Moving Past the Classic Model of Language Neurobiology. Brain Lang..

[38] Ardila A., Lopez M.V. (1984). Transcortical Motor Aphasia: One or Two Aphasias?. Brain Lang..

[39] Tremblay P., Gracco V.L. (2009). Contribution of the Pre-SMA to the Production of Words and Non-Speech Oral Motor Gestures, as Revealed by Repetitive Transcranial Magnetic Stimulation (RTMS). Brain Res..

[40] Dick A.S., Garic D., Graziano P., Tremblay P. (2019). The Frontal Aslant Tract (FAT) and Its Role in Speech, Language and Executive Function. Cortex.

[41] Dick A.S., Mok E.H., Raja Beharelle A., Goldin-Meadow S., Small S.L. (2014). Frontal and Temporal Contributions to Understanding the Iconic Co-Speech Gestures That Accompany Speech. Hum. Brain Mapp..

[42] Katzev M., Tüscher O., Hennig J., Weiller C., Kaller C.P. (2013). Revisiting the Functional Specialization of Left Inferior Frontal Gyrus in Phonological and Semantic Fluency: The Crucial Role of Task Demands and Individual Ability. J. Neurosci..

[43] Pedersen P.M., Jørgensen H.S., Nakayama H., Raaschou H.O., Olsen T.S. (1995). Aphasia in Acute Stroke: Incidence, Determinants, and Recovery. Ann. Neurol..

[44] Rogić M., Deletis V., Fernández-Conejero I. (2014). Inducing Transient Language Disruptions by Mapping of Broca’s Area with Modified Patterned Repetitive Transcranial Magnetic Stimulation Protocol. J. Neurosurg..

[45] Deletis V., Rogić M., Fernández-Conejero I., Gabarrós A., Jerončić A. (2014). Neurophysiologic Markers in Laryngeal Muscles Indicate Functional Anatomy of Laryngeal Primary Motor Cortex and Premotor Cortex in the Caudal Opercular Part of Inferior Frontal Gyrus. Clin. Neurophysiol..

[46] Tremblay P., Gracco V.L. (2010). On the Selection of Words and Oral Motor Responses: Evidence of a Response-Independent Fronto-Parietal Network. Cortex.

[47] Tremblay P., Small S.L. (2011). Motor Response Selection in Overt Sentence Production: A Functional MRI Study. Front. Psychol..

[48] Bannur U., Rajshekhar V. (2000). Post Operative Supplementary Motor Area Syndrome: Clinical Features and Outcome. Br. J. Neurosurg..

[49] Chivukula S., Pikul B.K., Black K.L., Pouratian N., Bookheimer S.Y. (2018). Contralateral Functional Reorganization of the Speech Supplementary Motor Area Following Neurosurgical Tumor Resection. Brain Lang..

[50] Kemerdere R., de Champfleur N.M., Deverdun J., Cochereau J., Moritz-Gasser S., Herbet G., Duffau H. (2016). Role of the Left Frontal Aslant Tract in Stuttering: A Brain Stimulation and Tractographic Study. J. Neurol..

[51] Neef N.E., Bütfering C., Anwander A., Friederici A.D., Paulus W., Sommer M. (2016). Left Posterior-Dorsal Area 44 Couples with Parietal Areas to Promote Speech Fluency, While Right Area 44 Activity Promotes the Stopping of Motor Responses. Neuroimage.

[52] Neef N.E., Anwander A., Bütfering C., Schmidt-Samoa C., Friederici A.D., Paulus W., Sommer M. (2018). Structural Connectivity of Right Frontal Hyperactive Areas Scales with Stuttering Severity. Brain.

[53] Alario F.-X., Chainay H., Lehericy S., Cohen L. (2006). The Role of the Supplementary Motor Area (SMA) in Word Production. Brain Res..

[54] Smirni D., Turriziani P., Mangano G.R., Bracco M., Oliveri M., Cipolotti L. (2017). Modulating Phonemic Fluency Performance in Healthy Subjects with Transcranial Magnetic Stimulation over the Left or Right Lateral Frontal Cortex. Neuropsychologia.

[55] Mandelli M.L., Caverzasi E., Binney R.J., Henry M.L., Lobach I., Block N., Amirbekian B., Dronkers N., Miller B.L., Henry R.G. (2014). Frontal White Matter Tracts Sustaining Speech Production in Primary Progressive Aphasia. J. Neurosci..

[56] Vassal F., Boutet C., Lemaire J.-J., Nuti C. (2014). New Insights into the Functional Significance of the Frontal Aslant Tract: An Anatomo-Functional Study Using Intraoperative Electrical Stimulations Combined with Diffusion Tensor Imaging-Based Fiber Tracking. Br. J. Neurosurg..

[57] Corrivetti F., de Schotten M.T., Poisson I., Froelich S., Descoteaux M., Rheault F., Mandonnet E. (2019). Dissociating Motor-Speech from Lexico-Semantic Systems in the Left Frontal Lobe: Insight from a Series of 17 Awake Intraoperative Mappings in Glioma Patients. Brain Struct. Funct..

[58] Aron A.R. (2007). The Neural Basis of Inhibition in Cognitive Control. Neuroscientist.

[59] Aron A.R., Fletcher P.C., Bullmore E.T., Sahakian B.J., Robbins T.W. (2003). Stop-Signal Inhibition Disrupted by Damage to Right Inferior Frontal Gyrus in Humans. Nat. Neurosci..

[60] Aron A.R., Robbins T.W., Poldrack R.A. (2004). Inhibition and the Right Inferior Frontal Cortex. Trends Cogn. Sci..

[61] Favre E., Ballanger B., Thobois S., Broussolle E., Boulinguez P. (2013). Deep Brain Stimulation of the Subthalamic Nucleus, but Not Dopaminergic Medication, Improves Proactive Inhibitory Control of Movement Initiation in Parkinson’s Disease. Neurotherapeutics.

[62] Jahanshahi M. (2013). Effects of Deep Brain Stimulation of the Subthalamic Nucleus on Inhibitory and Executive Control over Prepotent Responses in Parkinson’s Disease. Front. Syst. Neurosci..

[63] Obeso I., Wilkinson L., Casabona E., Speekenbrink M., Luisa Bringas M., Álvarez M., Álvarez L., Pavón N., Rodríguez-Oroz M.C., Macías R. (2014). The Subthalamic Nucleus and Inhibitory Control: Impact of Subthalamotomy in Parkinson’s Disease. Brain.

[64] van Wouwe N.C., Pallavaram S., Phibbs F.T., Martinez-Ramirez D., Neimat J.S., Dawant B.M., D’Haese P.F., Kanoff K.E., van den Wildenberg W.P.M., Okun M.S. (2017). Focused Stimulation of Dorsal Subthalamic Nucleus Improves Reactive Inhibitory Control of Action Impulses. Neuropsychologia.

[65] Boehler C.N., Appelbaum L.G., Krebs R.M., Hopf J.M., Woldorff M.G. (2010). Pinning down Response Inhibition in the Brain--Conjunction Analyses of the Stop-Signal Task. Neuroimage.

[66] Wessel J.R., Aron A.R. (2017). On the Globality of Motor Suppression: Unexpected Events and Their Influence on Behavior and Cognition. Neuron.

[67] Cañas A., Juncadella M., Lau R., Gabarrós A., Hernández M. (2018). Working Memory Deficits After Lesions Involving the Supplementary Motor Area. Front. Psychol..

[68] Fiori S., Guzzetta A., Mitra J., Pannek K., Pasquariello R., Cipriani P., Tosetti M., Cioni G., Rose S.E., Chilosi A. (2016). Neuroanatomical Correlates of Childhood Apraxia of Speech: A Connectomic Approach. Neuroimage Clin..

[69] Ashtari M., Lencz T., Zuffante P., Bilder R., Clarke T., Diamond A., Kane J., Szeszko P. (2004). Left Middle Temporal Gyrus Activation during a Phonemic Discrimination Task. Neuroreport.

[70] Guenther F.H., Hickok G. (2015). Role of the Auditory System in Speech Production. Handb. Clin. Neurol..

[71] Kadis D.S., Goshulak D., Namasivayam A., Pukonen M., Kroll R., De Nil L.F., Pang E.W., Lerch J.P. (2014). Cortical Thickness in Children Receiving Intensive Therapy for Idiopathic Apraxia of Speech. Brain Topogr..

[72] Preston J.L., Molfese P.J., Mencl W.E., Frost S.J., Hoeft F., Fulbright R.K., Landi N., Grigorenko E.L., Seki A., Felsenfeld S. (2014). Structural Brain Differences in School-Age Children with Residual Speech Sound Errors. Brain Lang..

[73] Tkach J.A., Chen X., Freebairn L.A., Schmithorst V.J., Holland S.K., Lewis B.A. (2011). Neural Correlates of Phonological Processing in Speech Sound Disorder: A Functional Magnetic Resonance Imaging Study. Brain Lang..

[74] Grande M., Meffert E., Schoenberger E., Jung S., Frauenrath T., Huber W., Hussmann K., Moormann M., Heim S. (2012). From a Concept to a Word in a Syntactically Complete Sentence: An FMRI Study on Spontaneous Language Production in an Overt Picture Description Task. Neuroimage.

[75] Cavanna A.E., Trimble M.R. (2006). The Precuneus: A Review of Its Functional Anatomy and Behavioural Correlates. Brain.

[76] Liégeois F.J., Morgan A.T. (2012). Neural Bases of Childhood Speech Disorders: Lateralization and Plasticity for Speech Functions during Development. Neurosci. Biobehav. Rev..

[77] Shriberg L.D., Potter N.L., Strand E.A. (2011). Prevalence and Phenotype of Childhood Apraxia of Speech in Youth with Galactosemia. J. Speech Lang. Hear. Res..

[78] Wechsler D. (2012). Wechsler Preschool and Primary Scale of Intelligence.

[79] McLeod S., Harrison L.J., McCormack J. (2012). The Intelligibility in Context Scale: Validity and Reliability of a Subjective Rating Measure. J. Speech Lang. Hear. Res..

[80] Usai M.C., Viterbori P., Gandolfi E., Traverso L. (2017). FE-PS 2-6: Batteria per la Valutazione delle Funzioni Esecutive in età Prescolare.

[81] Valeri G., Stievano P., Ferretti M., Mariani E., Pieretti M. (2015). BAFE Batteria per l’Assessment Delle Funzioni Esecutive in Età Prescolare.

[82] Conti E., Retico A., Palumbo L., Spera G., Bosco P., Biagi L., Fiori S., Tosetti M., Cipriani P., Cioni G. (2020). Autism Spectrum Disorder and Childhood Apraxia of Speech: Early Language-Related Hallmarks across Structural MRI Study. J. Pers. Med..

[83] Fischl B. (2012). FreeSurfer. Neuroimage.

[84] Jenkinson M., Beckmann C.F., Behrens T.E.J., Woolrich M.W., Smith S.M. (2012). FSL. Neuroimage.

[85] Tournier J.-D., Calamante F., Gadian D.G., Connelly A. (2004). Direct Estimation of the Fiber Orientation Density Function from Diffusion-Weighted MRI Data Using Spherical Deconvolution. Neuroimage.

[86] Biagi L., Lenzi S., Cipriano E., Fiori S., Bosco P., Cristofani P., Astrea G., Pini A., Cioni G., Mercuri E. (2021). Neural Substrates of Neuropsychological Profiles in Dystrophynopathies: A Pilot Study of Diffusion Tractography Imaging. PLoS ONE.

[87] Horbruegger M., Loewe K., Kaufmann J., Wagner M., Schippling S., Pawlitzki M., Schoenfeld M.A. (2019). Anatomically Constrained Tractography Facilitates Biologically Plausible Fiber Reconstruction of the Optic Radiation in Multiple Sclerosis. NeuroImage Clin..

[88] Broce I., Bernal B., Altman N., Tremblay P., Dick A.S. (2015). Fiber Tracking of the Frontal Aslant Tract and Subcomponents of the Arcuate Fasciculus in 5–8-Year-Olds: Relation to Speech and Language Function. Brain Lang..

[89] Hayes A.F. Introduction to Mediation, Moderation, and Conditional Process Analysis: Third Edition: A Regression-Based Approach. https://www.guilford.com/books/Introduction-to-Mediation-Moderation-and-Conditional-Process-Analysis/Andrew-Hayes/9781462549030.

[90] Chilosi A.M., Comparini A., Scusa M.F., Orazini L., Forli F., Cipriani P., Berrettini S. (2013). A Longitudinal Study of Lexical and Grammar Development in Deaf Italian Children Provided With Early Cochlear Implantation. Ear Hear..

[91] Chilosi A.M., Cipriani P. (2005). Tcgb-Test Di Comprensione Grammaticale Per Bambini.

[92] Bishop D.V.M., Giunti O.S. (2009). TROG-2: Test for Reception of Grammar, Version 2: Manuale/Dorothy M. V. Bishop.

[93] Vicari S., Luigi M., Alessandra L. (2007). TFL Test Fono-Lessicale.

[94] Dunn L.M., Pizzoli C., Tressoldi P.E. (2000). Peabody Picture Vocabulary Test-Revised (PPVT-R).

[95] Brizzolara D. (1989). Test Di Vocabolario Figurato. Technical Report of the Research Project 500.4/62.1/1134 Supported by a Grant from the Italian Department of Health to IRCCS Stella Maris.

[96] Hertrich I., Dietrich S., Ackermann H. (2016). The Role of the Supplementary Motor Area for Speech and Language Processing. Neurosci. Biobehav. Rev..

[97] Iuzzini-Seigel J. (2019). Motor Performance in Children With Childhood Apraxia of Speech and Speech Sound Disorders. J. Speech Lang. Hear. Res..

[98] Baddeley A. (2007). Working Memory, Thought, and Action.

[99] Morris N., Jones D.M. (1990). Memory Updating in Working Memory: The Role of the Central Executive. Br. J. Psychol..

[100] Kim H.-J., Choi S.Y., Ha J.-W. (2015). Speech-Motor Program/Programming in Children with Childhood Apraxia of Speech, Children with Articulatory and Phonological Disorders and Typically Developing Children. Commun. Sci. Disord..

[101] Case J., Grigos M.I. (2016). Articulatory Control in Childhood Apraxia of Speech in a Novel Word-Learning Task. J. Speech Lang. Hear. Res..

[102] Edeal D.M., Gildersleeve-Neumann C.E. (2011). The Importance of Production Frequency in Therapy for Childhood Apraxia of Speech. Am. J. Speech Lang. Pathol..

[103] Maas E., Gildersleeve-Neumann C., Jakielski K.J., Stoeckel R. (2014). Motor-Based Intervention Protocols in Treatment of Childhood Apraxia of Speech (CAS). Curr. Dev. Disord. Rep..

[104] Thomas D.C., McCabe P., Ballard K.J. (2014). Rapid Syllable Transitions (ReST) Treatment for Childhood Apraxia of Speech: The Effect of Lower Dose-Frequency. J. Commun. Disord..

[105] Spaulding T.J. (2010). Investigating Mechanisms of Suppression in Preschool Children With Specific Language Impairment. J. Speech Lang. Hear. Res..

[106] Marton K., Schwartz R.G. (2003). Working Memory Capacity and Language Processes in Children With Specific Language Impairment. J. Speech Lang. Hear. Res. JSLHR.

[107] Archibald L.M.D., Gathercole S.E. (2006). Short-Term and Working Memory in Specific Language Impairment. Int. J. Lang. Commun. Disord..

[108] Im-Bolter N., Johnson J., Pascual-Leone J. (2006). Processing Limitations in Children with Specific Language Impairment: The Role of Executive Function. Child Dev..

[109] Miller M.R., Giesbrecht G.F., Müller U., McInerney R.J., Kerns K.A. (2012). A Latent Variable Approach to Determining the Structure of Executive Function in Preschool Children. J. Cogn. Dev..

[110] Rossi E., Cheng H., Kroll J.F., Diaz M.T., Newman S.D. (2017). Changes in White-Matter Connectivity in Late Second Language Learners: Evidence from Diffusion Tensor Imaging. Front. Psychol..

[111] Takebayashi T., Marumoto K., Takahashi K., Domen K. (2018). Differences in Neural Pathways Are Related to the Short- or Long-Term Benefits of Constraint-Induced Movement Therapy in Patients with Chronic Stroke and Hemiparesis: A Pilot Cohort Study. Top. Stroke Rehabil..

[112] Tataranno M.L., Claessens N.H.P., Moeskops P., Toet M.C., Kersbergen K.J., Buonocore G., Išgum I., Leemans A., Counsell S., Groenendaal F. (2018). Changes in Brain Morphology and Microstructure in Relation to Early Brain Activity in Extremely Preterm Infants. Pediatr. Res..

[113] Fiori S., Pannek K., Podda I., Cipriani P., Lorenzoni V., Franchi B., Pasquariello R., Guzzetta A., Cioni G., Chilosi A. (2021). Neural Changes Induced by a Speech Motor Treatment in Childhood Apraxia of Speech: A Case Series. J. Child Neurol..

[114] Catani M., Mesulam M.M., Jakobsen E., Malik F., Martersteck A., Wieneke C., Thompson C.K., Thiebaut de Schotten M., Dell’Acqua F., Weintraub S. (2013). A Novel Frontal Pathway Underlies Verbal Fluency in Primary Progressive Aphasia. Brain.

[115] Mars R.B., Piekema C., Coles M.G.H., Hulstijn W., Toni I. (2007). On the Programming and Reprogramming of Actions. Cereb. Cortex.

[116] Shriberg L.D., Aram D.M., Kwiatkowski J. (1997). Developmental Apraxia of Speech: II. Toward a Diagnostic Marker. J. Speech Lang. Hear. Res..

